# Screening of Intron 1 Inversion of the Factor VIII Gene in 130 Patients with Severe Hemophilia A from a Pakistani Cohort

**DOI:** 10.4274/tjh.2017.0031

**Published:** 2017-08-02

**Authors:** Azhar Sattar, Shabbir Hussain, Muhammad Ikram Ullah, Saqib Mahmood, Shahida Mohsin

**Affiliations:** 1 University of Health Sciences Faculty of Medicine, Department of Hematology, Lahore, Pakistan; 2 University of Health Sciences Faculty of Medicine, Department of Biochemistry, Lahore, Pakistan; 3 Quaid-i-Azam University Faculty of Biological Sciences, Department of Biochemistry, Islamabad, Pakistan; 4 University of Health Sciences of Medicine, Department of Human Genetics and Molecular Biology, Lahore, Pakistan

**Keywords:** Hemophilia A, FVIII gene, Intron 1, Polymerase chain reaction, Pakistan

## To The Editor,

Hemophilia A (HA) is an X-linked bleeding disorder caused by diverse mutations in the factor VIII (FVIII) gene [1]. The causative mutations of the FVIII gene in severe hemophilia are very heterogeneous and spontaneous [2]. Inversion error in intron 1 occurs during male gametogenesis [3]. The incidence of FVIII mutations has been reported to vary in intron 1 (1%-5%) and intron 22 (50%) in people with hemophilia. Inversion mutations are responsible in 40%-60% cases of severe HA. Previous studies suggested that the prevalence of intron 1 inversion is variable, ranging from 1% to 5% in different countries [2,4]. The aim of this study was to investigate the prevalence of FVIII intron 1 inversion in patients with severe HA in Pakistan by polymerase chain reaction (PCR). According to the published data, this is the first report from a Pakistani population about the screening of intron 1 of the FVIII gene.

We selected 130 unrelated/sporadic severe HA patients referred to the Hemophilia Welfare Society of Pakistan after receiving ethical approval from the University of Health Sciences, Lahore, Pakistan. PCR amplification of genomic DNA was performed by using specific sets of primers as given in Table 1. The PCR reactions were conducted in 25-µL PCR reaction tubes. In the first step, initial denaturation of DNA was performed at 95 °C for 5 min. The second step consisted of 30 cycles of denaturation and annealing at temperatures of 94 °C and 58-65 °C for 1 min for each step and an extension at 72 °C for 2 min (according to the optimum annealing temperature of the primer, as given in Table 1). The third step consisted of final extension at 72 °C for 5 min. After amplification, products were resolved on 2% agarose gel with a 1-kb DNA ladder (GeneGauge, Caisson Laboratories) and visualized by UV trans-illuminator (Gel Doc EZ System, Bio-Rad). Interpretation of the gel bands was done according to standard band resolution.

**Figure 1:**
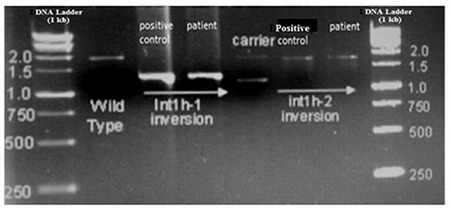
Molecular analysis of intron 1 inversion of the factor VIII gene in hemophilia A patients. A) The top panel shows normal results and the lower panel shows the patients with inversion. Diagram of normal DNA (top) and inversion (bottom) showing the location and orientation of relevant sequences as well as polymerase chain reaction primers and reactions (thin bars). B) Amplification of Int1h-1 using 9cR, 9F, and Int1h-2F primers (lanes 1, 2, and 3) and Int1h-2 using Int1h-2F, Int1h-2R, and 9F primers (lanes 4, 5, and 6). Control and inversion are males while the carrier is female.

Molecular analysis of inv1 of the FVIII gene in 130 patients with severe HA showed positive inversion only in 1 HA case with a frequency of 0.77% in our population. The mother of the patient was a carrier of the intron 1 inversion (Figure 1). The inversion-positive child had a familial HA history, with a typical band pattern in each reaction. At the age of 1 year, the child was diagnosed with hemophilia after excessive bleeding during a circumcision procedure. Factor VIII-C was 0.8 with no history of inhibitor against factor VIII. Previously, various studies explored the frequency of inv1 of FVIII in different people with hemophilia through PCR methods [1,5]. The frequency of inv1 reported in different populations is quite variable at 1.7% for the Italian population [6], 5.1% in the Spanish population [7], and about 8% in patients of Indian origin [2]. However, inv1 was not identified in 104 Hungarian patients with severe HA [8].

Intron 1 inversion is considered to be an important molecular factor, alteration of which may result in severe HA. Our results (0.77%) clearly indicated the variability between populations and confirm that inv1 is much less frequent than inv22.

## Figures and Tables

**Table 1:**
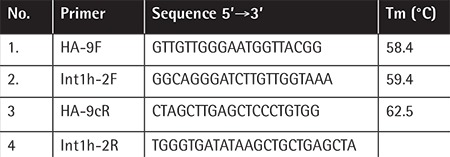
Primer sequences and their amplification conditions.
